# *In vitro* description of a new technique for stapled side-to-side jejunocecal anastomosis in horses and CT scan anatomical comparison with other techniques

**DOI:** 10.1186/1746-6148-10-S1-S9

**Published:** 2014-07-07

**Authors:** Marco Gandini, Gessica Giusto, Bryan Iotti, Alberto Valazza, Federica Sammartano

**Affiliations:** 1 Department of Veterinary Sciences, University of Turin, Via L. da Vinci 44, Grugliasco (TO), Italy

## Abstract

**Background:**

Stapled jejunocecal anastomoses are commonly performed in equine abdominal surgery. They carry higher complication rates compared to handsewn techniques. In human surgery various causes likely to lead to failure of stapled techniques have been evaluated, including staple line failure. Recently Freeman proposed a technique to perform a stapled jejunocecal anastomosis in horses while avoiding blind pouch formation. The aim of this study is to describe a method for stapled side-to-side jejunocecal anastomosis in horses and to compare it with other techniques with computed tomography to assess stomal area, shape and blind pouch size.

**Methods:**

Intestinal specimens comprising the cecum, ileum and jejunum from 18 horses were collected and were divided into three groups. In Group S a standard stapled side-to-side jejunocecal anastomosis was performed. In Group F the anastomosis was performed using a modified technique proposed by Freeman. In Group G the anastomosis was performed with a modified technique proposed by the authors. Inflated bowel segments were CT scanned to obtain a MultiPlanar Reconstruction of the stoma and afferent small intestine before calculating the cross-sectional area of each of these regions. The ratio of the measured areas was compared between the three techniques. The volume of the blind-end pouch was measured and its ratio with the intestinal area compared between techniques. The cecum was opened and the length of the stoma measured with a caliper and compared to the intended initial length.

**Results:**

The stomal/intestinal area ratio was not significantly different between techniques.

No statistically significant difference was found in the stomal ideal/real perimeter ratio.

There was no statistically significant difference in the intended/real stomal length ratio, and all techniques featured an increase in stomal length ranging from 2 to 12 %. Blind pouch formation was a consistent finding in Group S and was virtually absent in Groups F and G.

**Conclusions:**

Both the Freeman and the new (G) technique were comparable to the standard technique in terms of stomal area, stomal shape and difference in stomal elongation. They consistently produced a smaller blind pouch and allowed easier placement of the staplers.

## Background

Side-to-side jejunocecal anastomoses are commonly performed in equine abdominal surgery. Both handsewn and stapled techniques have been fully described [1], and have not changed significantly in the last 15-20 years, despite presence of higher complication rates compared to other types of intestinal anastomoses[1-3]. According to some authors stapled anastomoses carry higher risk of postoperative colic, reflux and repeat celiotomy compared to handsewn techniques, despite achieving the same survival rate [1,2]. The causes of these higher complication rates have not been fully evaluated. In human gastrointestinal surgery there is an ongoing debate about the effectiveness and safety of stapled techniques, in comparison to handsewn techniques [4,5]. In human medicine various causes likely to lead to failure of stapled techniques have been evaluated and include staple line failure, stricture and anastomotic leakage [4-6]. All these events could also occur in horses, alone or in association with the formation of a blind pouch, a complication of current techniques of jejunocecal side-to-side anastomoses, either handsewn or stapled [1,7]. A blind pouch forms when the portion of the proximal segment that extends distal to the stoma is excessively long, leading to stasis of intestinal content, abnormal bacterial growth, impaction, ulceration or perforation [8-18].

Recently Freeman proposed a new technique to perform a stapled jejunocecal anastomosis in horses while avoiding blind pouch formation, although the main focus of his work was to avoid complications caused by the stapler insertion sites [2]. The site of stapler insertion is an issue of great importance [2] because, if the enterotomy sites chosen to insert the stapler's arms are too close to the jejunal stump end, the stoma will be shorter than the length of the stapler. If, instead, they are too oral, they could result in the formation of a long blind pouch [1,2]. Stomal size and shape could also play a significant role in the outcome of this technique [1,2] and attempting to achieve a stomal size that is similar to that of the afferent distal jejunum has been proposed as the ideal outcome [2] .

The purpose of the present study is to anatomically compare the standard stapled technique (S) with the technique described by Freeman (F) and a proposed modified technique (G) for stapled side-to-side jejunocecal anastomosis in terms of stomal area, stomal shape and blind pouch size.

## Methods

Intestinal specimens comprising the cecum, ileum and three meters of jejunum from 18 horses (mean age 24 months, range 18-30 months, mean weight 450, range 420-480) without clinically evident intestinal disease were collected immediately after death at the Didactical Abattoir, Department of Veterinary Sciences, University of Turin, and maintained in warm Ringer lactate solution until testing, that was completed within 6 hours from collection. Bowel segments were divided into three groups of 6 specimens each: Group S underwent a standard stapled jejunocecal anastomosis [1]. Group F received a stapled jejunocecal side-to-side anastomosis with a modified technique described by Freeman [2]. Group G received a stapled jejunocecal side-to-side anastomosis performed with a modified technique. All anastomoses were performed with an Autosuture Multifire GIA 80 Linear Cutting Stapler (Covidien Italia, Segrate, Milano, Italy).

### Surgical techniques

All anastomoses were performed by the same surgeon (MG) and the same assistant (GG) in order to reduce variability between the techniques. All anastomoses were placed between the dorsal and the medial band of the cecum, level with the cecocolic fold [1], approximately 20 cm from the ileocecal valve. In the standard technique (Group S) the jejunal stump was closed at its end with a Parker-Kerr pattern oversewn by a Cushing pattern with 2-0 polydioxanone (PDS II, , Ethicon, J&J Italia, Milano, Italy). Two stay sutures were placed 5-8 mm deep to the antimesenteric Border of the jejunum [1] to approximate it to the cecal wall. An enterotomy was performed in each of the jejunum and cecum orad to the intended site of the stoma, to allow insertion of the two arms of the linear cutting stapler. The stapler was then closed and fired. The enterotomies were closed with a continuous Cushing pattern with 2-0 Polydioxanone (PDS II, Ethicon, J&J Italia, Milano, Italy). The staple line was not oversewn. The technique described by Freeman (Group F) was performed as previously described [2]. The jejunal stump was not closed at its end. It was opposed on the body of the cecum with stay sutures. An enterotomy was made in the cecum distally to the intended stoma site. The linear cutting stapler’s arms were inserted in the open jejunal stump and through the cecal enterotomy. The stapler was then closed and fired. The open end of the jejunal stump was partially closed with a Cushing pattern for approximately half its length with 3-0 polydioxanone (PDS II, Ethicon, J&J Italia, Milano, Italy) starting from the mesenteric side. When the remaining opening of the jejunal stump was nearly same size as the enterotomy on the cecal body, the suture was tied. One edge of the cecal enterotomy was then sutured to the edge on the same side of the jejunal stump with a continuous suture. The same was done on the other side. This caused the formation of a Y-shaped closure. Sutures were then oversewn with a Cushing pattern. Staple lines were not oversewn but two reinforcing sutures were placed around the anastomosis, as described [2].

**Figure 1:**
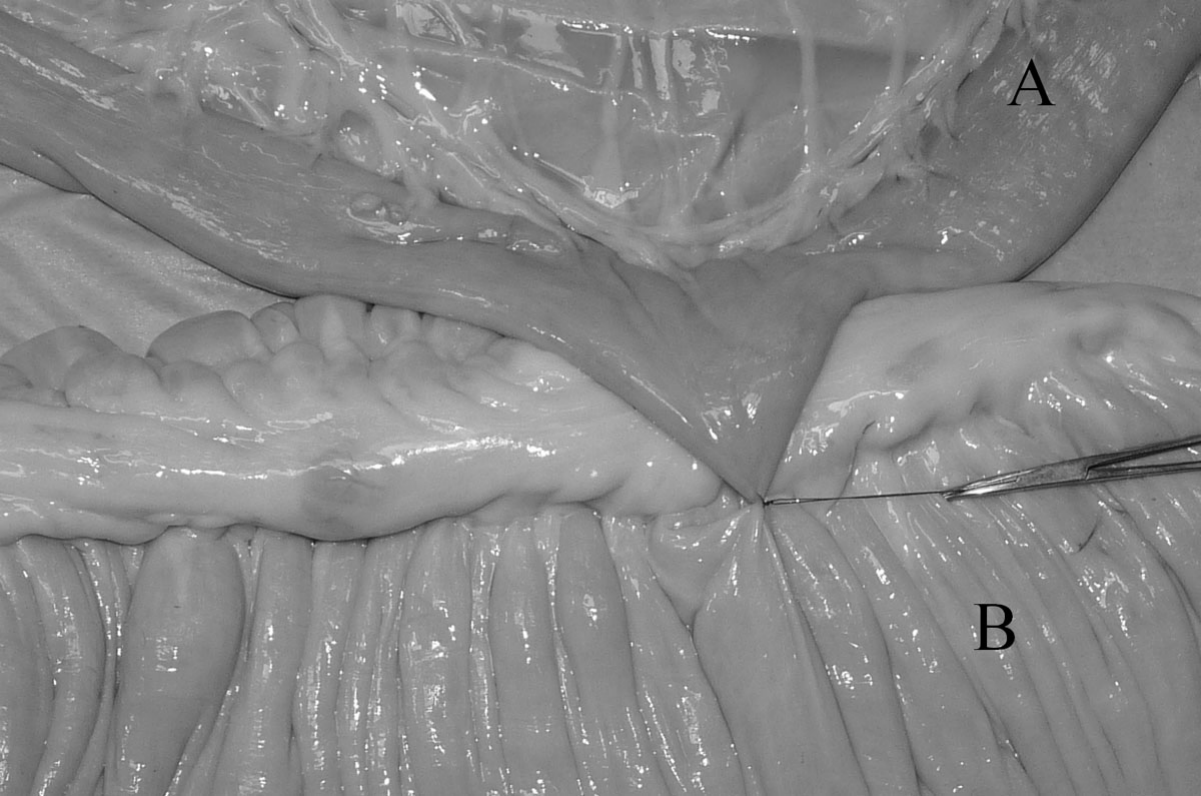
Stay suture placed slightly deep in the antimesenteric side of the jejunum (A) and joining it to the cecal body (B)

**Figure 2:**
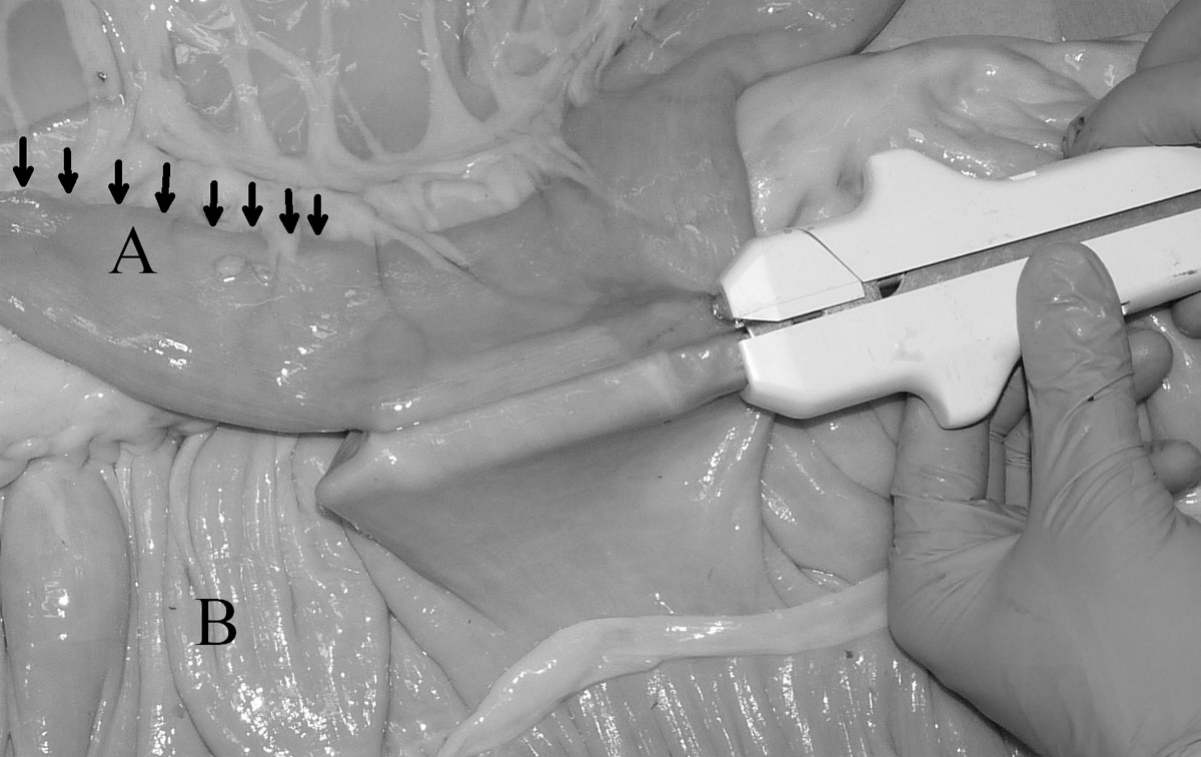
**Placement of the stapler with the modified anvil tip inside the jejunal stump.** Note the length of jejunum left distally to the anastomotic site (black arrows). This will be removed afterwards.

**Figure 3:**
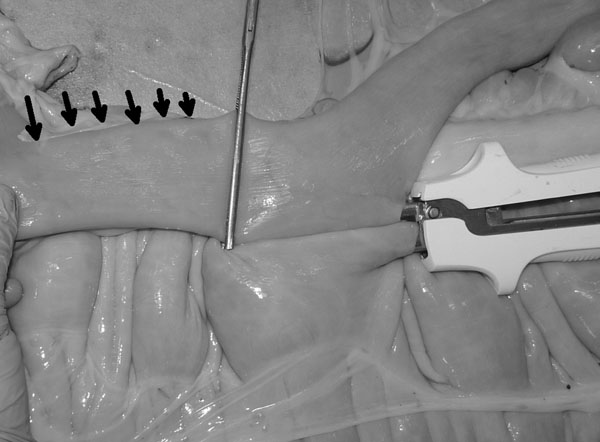
**Hartmann’s crushing clamp in place.** At this stage the portion of jejunum distal to the clamp (black arrows) will be removed.

**Figure 4:**
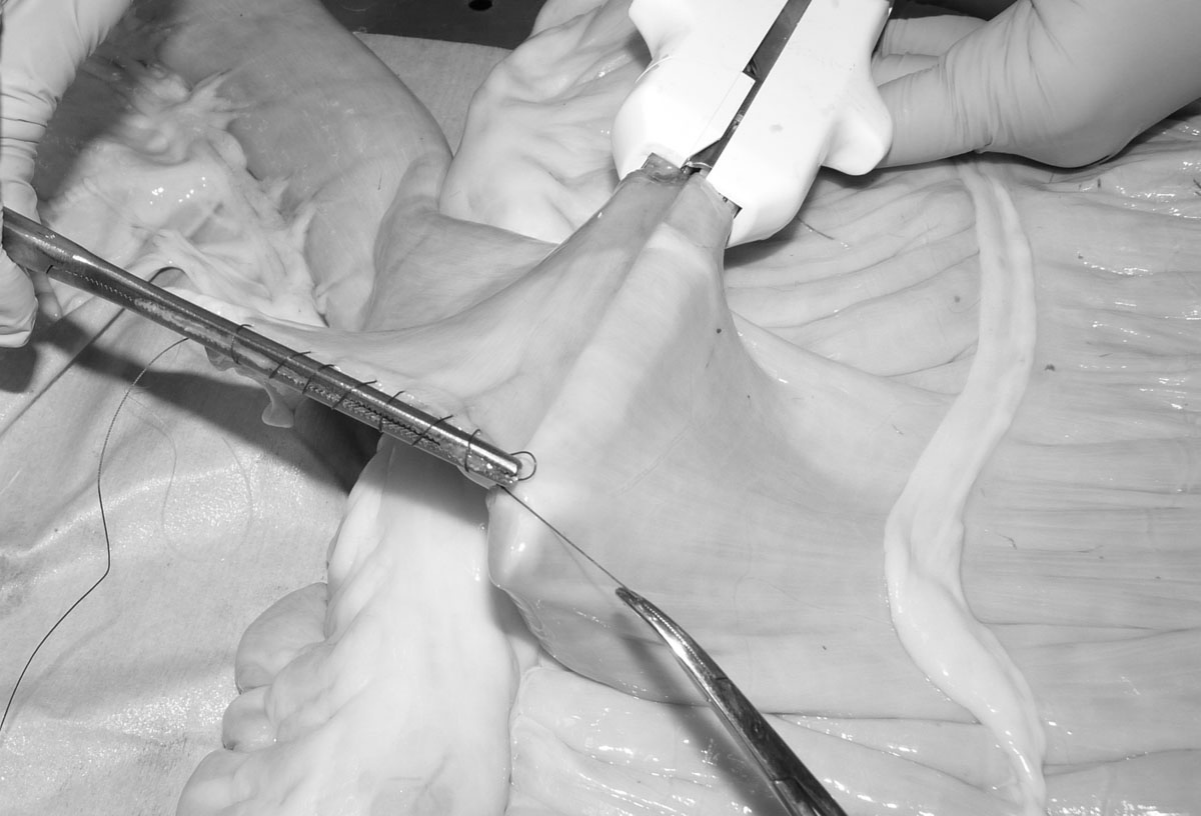
Parker-Kerr suture started from the cecal body (B) and continued towards the mesenteric side of the jejunum (A).

In the modified technique (Group G) the jejunum was not cut. It was opposed to the cecal body wall with one stay suture placed 5 mm deep [1] to the antimesenteric border (Fig. 1). The stay suture was clamped in a mosquito forceps and held by an assistant. One enterotomy, approximately 1.5 cm long, was performed just distal to the stay suture and 5 mm deep [1] to the antimesenteric border of the jejunum and in the cecal body. The anvil arm of an Autosuture Multifire Gia 80 was modified by removing the plastic beveled tip (Fig. 2) and the stapler was then introduced through the previously made enterotomies with the anvil arm into the jejunum. The stapler was closed and fired. A Hartmann crushing clamp was placed on the jejunum just distal to the anvil arm tip and the distal jejunum was resected (Fig. 3). A Parker-Kerr suture with 2-0 polydioxanone (PDS II, , Ethicon, J&J Italia, Milano, Italy) was then started from the body of the cecum and continued towards the mesenteric side of the jejunum (Fig. 4) to invert the distal edge of the jejunum. The clamp was removed and the jejunal stump accordioned. An oversewing Cushing pattern was then placed towards the cecal body wall. At the mesenteric border of the jejunum, the bites were placed further back (approximately 8 mm) from the inverted edge of the jejunum compared to their position as the suture line progressed towards the cecum (approx 2-3 mm). This created a beveled edge that angled distally from the mesenteric side of the jejunum to the cecum, so the jejunum formed an angle of less than 90° with the cecum. The suture was then tied onto the cecal body. Finally the stapler was removed and the enterotomies closed with a continuous inverting suture. The staple line was not oversewn and no reinforcing sutures were applied.

Bowel segments were then connected to a manometer (MRT-9835, Logiko, Ausilium, Beinasco, Torino, Italy) as previously described [19] and inflated by means of a compressor (C240-10, Gentilin , Trissino, Vicenza, Italy) providing 1 l/min of air at a pressure of 8 mmHg. Said pressure was maintained while the specimen wassubmitted to CT scanning [7]. During previous work [20] we found that modifications in the shape of the stoma occur at pressures of up to 8 mmHg, then subside, having exhausted the compliance of the staple line and of tissues involved in the formation of the stoma. After CT scanning the cecum was cut open and the actual stomal length measured with a caliper.

### Image acquisition, MultiPlanar Reconstructions, stomal area and perimeter, proximal jejunal area and perimeter, blind pouch volume and distal jejunal area

The images were acquired using a single slice Computed Tomography (CT) unit in axial mode using a slice thickness of 1mm, a matrix size of 512x512, a medium smooth reconstruction algorithm (defined as CHST by the CT software), 120kVp and 130mAs. The images were transferred to the visualization workstation and viewed with standardized windowing parameters (WW 970, WL -414.50). The Osirix software was then used to calculate the volume and area of the blind pouch. Three-dimensional MultiPlanar Reconstructions (MPR) of the stoma were exported as 16-bit Monochrome DICOM (Digital Imaging and Communication in Medicine) lossless images, for use in the ImageJ measurement software. Four stomas were not monoplanar and required the use of thick-slice MPR reconstructions using volume rendering (MPR slice thickness range: 8.19 mm to 38.12mm, mean±SD: 19.06±11.87). Using the wand tool (tolerance 100, legacy mode) the stomal area and perimeter were calculated on the MPR images, while the proximal jejunal area and perimeter were calculated on the original images.

### Data analysis –Stomal/intestinal ratio

For each specimen, a ratio was obtained by dividing the stomal area by the jejunal area.

### Data analysis – stomal ideal/real perimeter ratio

We calculated the perimeter of an ideal circle of the same area as the stoma and then obtained a ratio by dividing this ideal value by the real one. This value gives an indication of the shape of the stoma. A ratio of 1 indicates a perfectly circular stoma.

### Data analysis - variation between real stomal length and intended initial stomal length

After the scan was completed, the cecum was opened at a site distant from the anastomosis and the effective length of the stoma was measured with a caliper and compared to the intended initial length. The intended initial stomal length is the length of the stoma that the procedure should have formed based on the choice of the length of the stapler. A percentage of variation was obtained by comparing the length of the stoma measured directly with a caliper on the specimen at the time of incision and after deflation following image acquisition.

### Data analysis- blind pouch volume/area ratio

The volume of the blind pouch was calculated in Osirix by 2D region growing followed by ROI volume calculation using a PowerCrust algorithm. The resulting value was then divided by the area of the afferent jejunum.

### Statistical analysis – descriptive statistics

Using the R software (version 2.15.1 32-bit) we measured the normality of each group using a Shapiro-Wilk normality test and obtained means, standard deviation, median and interquartile ranges.

### Statistical analysis – inferential statistics

Our work aims to ascertain the presence of statistically significant differences between various parameters of the anastomoses performed using these three techniques. The considered parameters were blind pouch volume/area ratio, stomal/jejunal area ratio, ideal/real perimeter ratio and percentage of stomal length variation.

We used Pairwise Student's T-Test on normally distributed data (Stomal ideal/real perimeter ratio) and Pairwise Wilcoxon's Rank Sum Test on non-normally distributed data (Stomal/intestinal area ratio, Intended/real stomal length, Blind pouch volume/area ratio) with significance set for p< 0.05.

## Results

Results are summarized in Table 1.

**Table 1:** Mean±SD (median) of different parameters tested. p<0.05, different letters indicate statistically significant difference

	Stomal/intestinal area ratio	Stomal ideal/real perimeter ratio	Intended/real stomal length ratio (%)	Blind pouch volume/area ratio
**Group S**	1.23±0.68 (1.16)	0.88±0.03 (0.88)	8±3 (6)%	1.46±0.52 (1.24)^b^

**Group F**	0.95±0.28 (0.85)	0.83±0.06 (0.85)	2±10 (6)%	0.13±0.17(0.07)^a^

**Group G**	1.23±1.04 (1.16)	0.84±0.1 (0.87)	8±3 (7)%	0.27±0.22 (0.33)^a^

**Figure 5:**
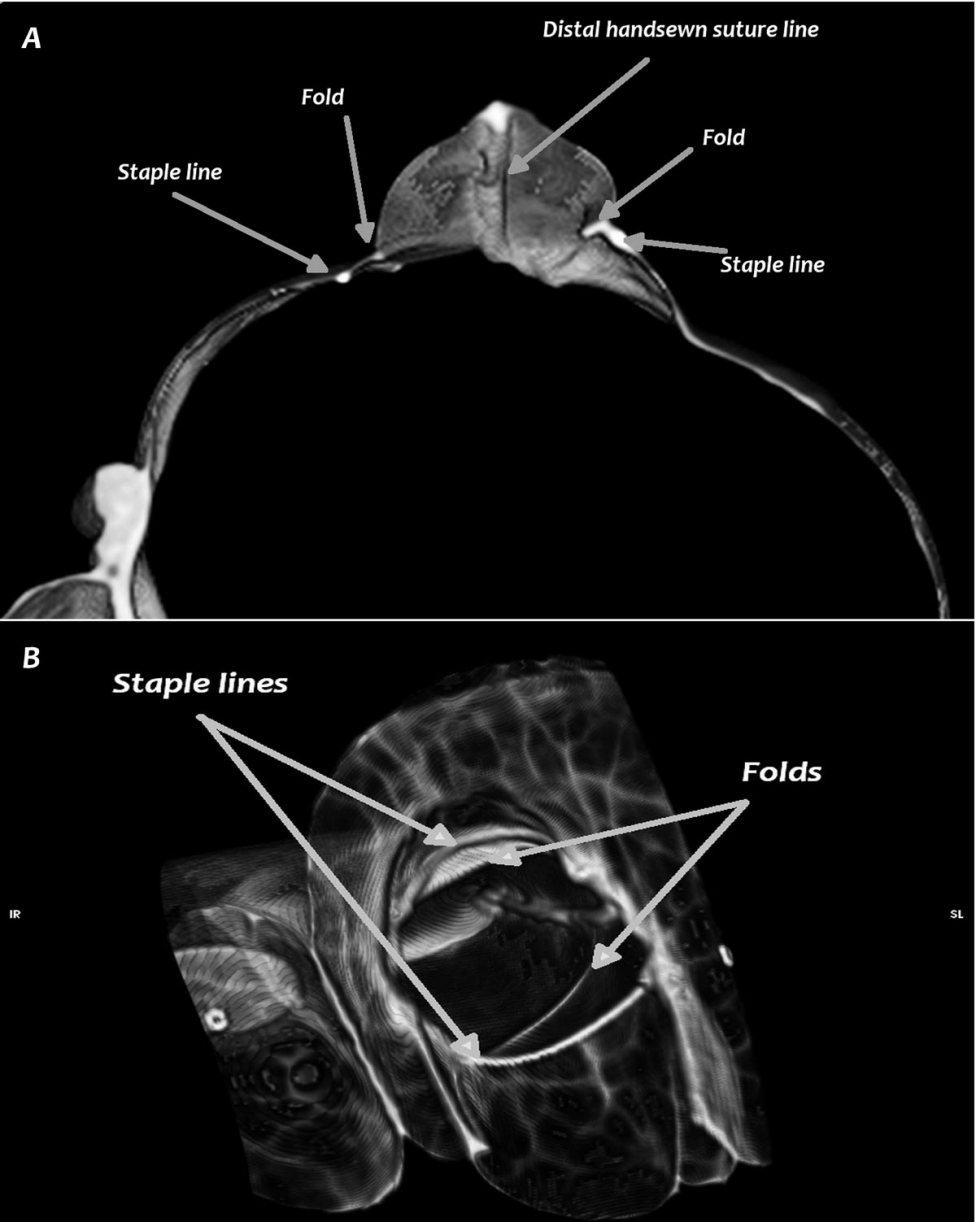
**CT scan of the Freeman technique.** Note the two folds of tissue in the jejunal wall inside the stoma, not corresponding to staple lines. A) transverse view, B) 3D reconstruction of the anastomosis, viewed from inside the cecum.

The stomal/intestinal area ratio was not significantly different between techniques. No statistically significant difference was found in the stomal ideal/real perimeter ratio, although there was a trend towards a more circular shape in Group S. The technique in Group F resulted in the formation, upon inflation, of two folds in the jejunal wall placed internally to each row of staples (Fig. 5). There was no statistically significant difference in the intended/real stomal length ratio, and all techniques resulted in an increase in stomal length ranging from 2 to 12 % of the intended value. Only two cases, both in Group F, resulted in a decrease in stomal length. Blind pouch formation was a consistent finding in Group S. There was a statistically significant difference in the blind pouch volume/area ratio between Group F and Group S (p=0.02) and between Group G and Group S (p=0.01). There was no statistically significant difference between Group F and Group G.

## Discussion

Jejunocecal anastomoses in horses carry a higher risk of complications than other anastomotic techniques and, amongst them, stapled anastomoses carry higher complication rates compared to handsewn techniques [2]. For this reason there is a need to better understand the pitfalls of commonly used techniques, so that they may be improved. This surgical technique could be sensitive to minor changes [2]. It is therefore necessary to carefully and thoroughly evaluate new modifications as they are introduced.

In our study we proposed a modified stapled technique and compared it *in vitro* to two other previously described techniques from an anatomical point of view. When compared to the handsewn technique, the modified version proposed by Freeman [2] is reported to have higher complication rates while featuring the same survival rate. The author supposed this higher complication rate was caused by the formation of a wider stoma, among other possible causes [2], In our study this technique produced a stoma similar to or narrower than the other two techniques, due to the formation of two folds of tissue in the jejunal wall lying internally to each row of staples (Fig. 5) and probably caused by the Y shaped closure. Narrowing the stoma may not necessarily lead to problems [7], although the behaviour of these folds *in vivo* cannot be easily established. The stomal/intestinal area ratio was higher in Group F than with the other two techniques, demonstrating that an 80 mm long stapler is able to produce a stoma as wide as the proximal intestine feeding into it in 400 kg horses. Also, there was no statistically significant difference in the stomal ideal/real perimeter ratio, although there was a trend to a more “circular” shape in Group S.

By producing the two folds axially to the staple line, the F technique had a lower ideal/real perimeter ratio when compared to the other techniques. Although the difference was not statistically significant, the formation of these folds could decrease stomal compliance to the passage of ingesta, possibly participating in increasing the complication rate of this technique.

All techniques exhibited an increase in the final stomal length, ranging from 2 to 12 % longer than the intended initial stomal length, and this datum is in accordance with other studies [7]. This finding must be taken into account by surgeons, who must be aware that the choice of an 80 mm long stapler can result in a final stomal length of up to 90mm, potentially due to the stab incision. In fact ,in Group F, where no stab incision was used to introduce the staplers, we noticed a decrease in stomal length in two cases, possibly caused by an excessive inversion of the oversewing distal sutures used to close the jejunal stump.

Blind pouch formation is a common complication of side-to-side anastomoses, both handsewn or stapled [2,8-18], that can cause obstruction of the afferent bowel [2], inflammation, haemorrhage, necrosis, and leakage [8-18]. Because it was not possible to exactly and consistently measure the length of the blind pouch, we initially measured its volume. Since the volume was influenced also by the diameter of the afferent jejunum, we deemed the introduction of the blind pouch volume/area ratio necessary to reduce inter-sample variation. Blind-end pouch formation was a consistent finding in the S technique, while it was not present in the F technique and strongly reduced in the G technique. The purpose of the F technique was to avoid problems with placing the stab incision in the jejunum either too close to or too far from the oversewn end. The former would create too short a stoma and the latter would create a blind pouch in the jejunum. The F technique allows full insertion of the stapler into the jejunum to create as large a stoma as possible. Preventing a blind pouch could therefore be up benefit of this technique. Although it does not seem related to a reduced complication rate, it could perhaps be related to a better survival rate [2]. In fact, short term complications could be caused by other issues (e.g. anastomotic leakage), while blind-end pouch formation could instead play a role in medium or long term complications [18].

In the G technique, cutting the jejunum and closing the stump after stapling also removed the problem of placing the enterotomies in the correct position for stapler insertion. This is a crucial point in performing a functional anastomosis. The same goal can certainly be accomplished by a second application and firing of the linear cutting stapler or using a linear stapler (e.g. Ta-90) transversely on the jejunal stump [21]. We preferred the use of the Parker-Kerr method to close the end of the jejunum because the stapled line could be difficult to accordion in order to reduce the blind pouch volume. We also proposed a further modification regarding the Parker-Kerr suture used to close the distal end of the jejunal stump. In this technique, larger bites are taken in the mesenteric portion of the suture while smaller bites are taken in the antimesenteric portion, producing a beveled shape in the jejunal stump.

We performed the modified Parker-Kerr suture while the stapler was still in place because it avoided straining the staple line by holding the two bowel segments together. Removing the stapler before performing the second suture could further reduce the formation of the blind end, but we think modifying the stapler’s anvil tip accomplished the same goal. If the anvil tip is not removed then the removal of the stapler soon after firing will be necessary in order to reduce the blind pouch. We had the impression that closing the distal end of the jejunum without the stapler supporting the staple line would put sufficient strain on the staple line to disrupt it, so we preferred to avoid this risk by leaving the stapler in place until completion of the Parker-Kerr suture.

The main advantage of the G technique versus the F technique is the possibility of placing the stapler in a proximo-distal direction. In fact, relative placement of the stoma onto the cecal body could be another issue that influences the performance of jejunocecal anastomoses. In the Freeman technique performed in vivo, having the surgeon insert the stapler disto-proximal might be a problem, possibly leading to an excessively apical placement of the stoma compared to the other techniques since the abdominal incision is in the way, forcing the stoma to be made too close to the apex. With the G and S techniques this is not an issue, although in our study this aspect might not have been fully evaluated because all the stoma were placed at the same distance from the ileocecal valve.

The oversewing of staple lines has been proposed in stapled anastomoses, although this operation could remove the advantage offered by this type of technique in terms of reduced surgical time [1,20], and possibly modify the stomal shape. We decided not to oversaw the staple lines because all the techniques have distal and proximal handsewn sutures that can strengthen these crucial locations.

Both the Freeman and the G techniques were comparable to the standard technique in terms of stomal area, shape and difference in stomal elongation. They consistently produced a significantly smaller blind pouch and made proper placement of the staplers easier. The latter might prove easier to perform *in vivo* compared to the former because of the proximo-distal insertion of the stapler. For these reasons the F and G techniques should be preferred to the standard technique, although further *in vivo* studies are needed.

## Competing interests

The authors declare that they have no competing interests

## Authors’ contributions

MG and GG developed the technique, studied the experimental protocol, performed the surgeries, participated in the CT acquisition, collected data, wrote and reviewed the paper.

BI participated in the CT acquisition, performed all the image data analysis and statistics, wrote and reviewed the paper.

FS and AV performed the CT acquisitions, participated in reviewing the paper.

## Declarations

No funding was obtained for this study

This article has been published as part of *BMC Veterinary Research* Volume 10 Supplement 1, 2014: Selected articles from the Eleventh International Equine Colic Research Symposium. The full contents of the supplement are available online at http://www.biomedcentral.com/bmcvetres/supplements/10/S1. Publication of this supplement has been funded by The British Equine Veterinary Association.
